# HIV-1 Enhancing Effect of Prostatic Acid Phosphatase Peptides Is Reduced in Human Seminal Plasma

**DOI:** 10.1371/journal.pone.0016285

**Published:** 2011-01-20

**Authors:** Julie A. Martellini, Amy L. Cole, Pavel Svoboda, Olga Stuchlik, Li-Mei Chen, Karl X. Chai, Bhushan K. Gangrade, Ole E. Sørensen, Jan Pohl, Alexander M. Cole

**Affiliations:** 1 Department of Molecular Biology & Microbiology, Biomolecular Science Center, Burnett School of Biomedical Sciences at the University of Central Florida College of Medicine, Orlando, Florida, United States of America; 2 Microchemical and Proteomics Facility, Emory University, Atlanta, Georgia, United States of America; 3 Biotechnology Core Facility Branch, Division of Safety Research, Centers for Disease Control and Prevention, Atlanta, Georgia, United States of America; 4 Center for Reproductive Medicine, Orlando, Florida, United States of America; 5 Division of Infection Medicine, Department of Clinical Sciences, Lund University, Lund, Sweden; Academic Medical Center, Netherlands

## Abstract

We recently reported that HIV-1 infection can be inhibited by innate antimicrobial components of human seminal plasma (SP). Conversely, naturally occurring peptidic fragments from the SP-derived prostatic acid phosphatase (PAP) have been reported to form amyloid fibrils called “SEVI” and enhance HIV-1 infection *in vitro*. In order to understand the biological consequence of this proviral effect, we extended these studies in the presence of human SP. PAP-derived peptides were agitated to form SEVI and incubated in the presence or absence of SP. While PAP-derived peptides and SEVI alone were proviral, the presence of 1% SP ablated their proviral activity in several different anti-HIV-1 assays. The anti-HIV-1 activity of SP was concentration dependent and was reduced following filtration. Supraphysiological concentrations of PAP peptides and SEVI incubated with diluted SP were degraded within hours, with SP exhibiting proteolytic activity at dilutions as high as 1∶200. Sub-physiological concentrations of two prominent proteases of SP, prostate-specific antigen (PSA) and matriptase, could degrade physiological and supraphysiological concentrations of PAP peptides and SEVI. While human SP is a complex biological fluid, containing both antiviral and proviral factors, our results suggest that PAP peptides and SEVI may be subject to naturally occurring proteolytic components capable of reducing their proviral activity.

## Introduction

Mucosal tissues express a number of antimicrobial peptides and proteins that exert broad spectrum activity against fungi, bacteria, and viruses such as HIV-1 [1]–[3]. Many are cationic in nature, and owe their ability to prevent microbial and viral infections in part to electrostatic interactions with membrane surfaces [4], [5]. The antimicrobial activity of human seminal plasma (SP) has been established for decades [6], [7]. Several reports have studied the individual ubiquitous innate immune components present in SP, including lactoferrin, lysozyme_,_ HBD-1, and antimicrobial chemokines [8], [9], as well as SP specific antimicrobial peptides, including HE2α C-terminal fragments [10], and semenogelin-derived peptides [11], [12]. While the antibacterial properties of seminal fluid have been established, only recently have the anti-HIV-1 activities of human SP been described [13].

While human SP contains various antimicrobial factors, a number of proviral factors have also been identified [14], [15]. One recent study has reported the ability of a natural proteolytic fragment of the protein prostatic acid phosphatase (PAP^286^; residues 248–286) to form amyloid fibrils that are capable of enhancing HIV-1 infection. Amyloid fibrils from the PAP-derived peptide were generated in vitro through long periods of agitation, and deemed Semen-derived Enhancer of VIrus (SEVI) [16]. Interestingly, the positive charge (pI = 10.2) of SEVI reportedly decreases the electrostatic repulsion between the negative charge of the HIV virions and the negative overall charge of the target cell membrane, leading to enhanced virion attachment [17].

Whole PAP is stored in the prostate, along with various other enzymes and a large zinc ion reservoir that maintains prostate-derived enzymes in an inactive state [18], [19]. Upon ejaculation, semen forms a gelatinous meshwork and is subsequently liquefied by activated prostatic enzymes, principally the kallikrein-like serine protease prostate-specific antigen (PSA) [20], [21]. At neutral pH, PAP reportedly exhibits amidolytic activity on semenogelins, the major components of the seminal coagulum [22]. We have recently reported that SP continues to degrade most of its intrinsic proteins after liquefaction [13]; however, it has yet to be determined how PAP undergoes cleavage into the PAP^286^ fragments.

In the current study, we sought to elucidate the biological role of PAP^286^ with respect to its ability to form amyloid fibrils and promote HIV-1 infection in the presence of human SP. While we could confirm that PAP-derived amyloid fibril formation exhibited HIV-1 enhancing activity, we found that this proviral activity was neutralized by human SP. Moreover, SP retained significant anti-HIV-1 activity in the presence of supra-physiological concentrations of PAP amyloid fibrils. PAP peptides were degraded into fragments that were not proviral within 3 h of incubation with diluted SP, and this proteolytic degradation was due to SP enzymes, including prostate derived PSA and prostasin [20], [23], and epithelially derived matriptase [24]. Together, these results confirm the ability of PAP-derived amyloid fibrils to enhance HIV-1 infection alone; however, in the physiological milieu these peptides and their resulting fibrils might be susceptible to proteolytic degradation that could inhibit their proviral activity.

## Materials and Methods

### Ethics Statement

Samples of human semen were collected by the Center for Reproductive Medicine as described previously [13]. As these samples were discarded from routine testing, and not linked to any identifiers, the University of Central Florida IRB has deemed them exempt human subjects; therefore obtaining informed consent was not applicable for this study.

### Processing of human seminal plasma and PAP peptides

Semen was collected by the Center for Reproductive Medicine as described previously [13]. Briefly, semen was collected from patients who were asked to refrain from ejaculation for 2–5 days prior. Semen was obtained via dry masturbation into a sterile polypropylene cup, and allowed to liquefy for 30 min at room temperature. A total of 103 individual seminal plasma (SP) samples were centrifuged for 30 min at 1500× g, and the supernatants were pooled and stored at −80°C or used for subsequent analyses. An aliquot of the SP pool, referred to as “SP+(Ab)” hereafter, was filtered through a sterile, nylon, 45 µm syringe filter (Fisher Scientific, Pittsburgh, PA USA) and supplemented with 100 units/ml penicillin, 100 µg/ml streptomycin, and 50 µg/ml gentamicin as described previously [16]. An additional pool of eight semen samples was collected, allowed to liquefy at room temperature for 30 min, and portioned into three fractions, “Pre-SP” (SP obtained from semen prior to freezing at −20°C), “Post-SP” (SP obtained from semen after freezing) and whole semen. All semen and SP samples were then stored at −80°C until analyzed.

The synthetic PAP peptides corresponding to fragments PAP 248–266 (PAP^266^) and PAP 248–286 (PAP^286^) were synthesized as previously described [16], via standard Fmoc solid phase chemistry using a model 433A peptide synthesizer (Applied Biosystems, Foster City, CA, USA). Crude peptides were purified by preparative reversed-phase HPLC to >95% purity, and were lyophilized. Masses were confirmed by MALDI-TOF MS. The purified synthetic peptides were resuspended in sterile PBS and stored at −20°C. To generate SEVI, peptides were agitated in a thermomixer at 1400 rpm for 18 h at 37°C as described previously [16]. For visual documentation, PAP peptides (1–5 mg/ml) with or without SP (1%) were agitated in a thermomixer at 1400 rpm for 36 h at 37°C to form observable amyloid fibrils as described in [16], [25], and were pulsed for 20 s at 10,000 rpm in a microcentrifuge to sediment amyloid fibrils. Amyloid fibril formation was monitored with Congo red staining as previously described [25]. Fibril formation was measured at OD_490nm_ using a spectrophotometer. Stained fibrils were visualized using phase contrast microscopy (Axiovert 200 M microscope, and Axiovision 4.5 software, Carl Zeiss MicroImaging, Inc., Thornwood, NY, USA).

### Cell lines and viruses

The HeLa-derived epithelia TZM-bl cell line and the lymphocytic PM1 cell line were obtained from the National Institutes of Health AIDS Research and Reference Reagent Program (Germantown, MD, USA), and Peripheral Blood Mononuclear Cells (PBMCs) were obtained from healthy donors by AllCells, LLC (Emeryville, CA, USA). TZM-bl cells were cultured in high glucose DMEM (Mediatech, Manassas, VA, USA) supplemented with 100 units/ml penicillin, 100 µg/ml streptomycin, and 10% (v/v) Fetal Bovine Serum (FBS) (Gemini Bio-Products, West Sacramento, CA, USA). PM1 cell cultures were maintained at a density of 0.4–0.8×10^6^ cells/ml in RPMI 1640 supplemented with 100 units/ml penicillin, 100 µg/ml streptomycin, 10 mM HEPES, and 20% (v/v) FBS. PBMCs were stimulated with phytohemagglutinin (PHA) (5 µg/ml) and 50 units/ml IL-2 for the first 3 days, and then maintained at a density of 0.75–1.5×10^6^ cells/ml in R10 medium (RPMI 1640 with 10% FBS) supplemented with 25 units/ml IL-2 (Roche Applied Science, Indianapolis, IN, USA). HIV-1 BaL, an R5-tropic strain, was obtained from the National Institutes of Health AIDS Research and Reference Reagent Program (Germantown, MD, USA). HIV-1 BaL was propagated in PM1 cells, and supernatant was filtered and stored at -80°C until needed. Viral quantification was achieved via a sensitive commercial ELISA for p24gag (PerkinElmer, Waltham, MA, USA).

### Antiviral and cytotoxicity assays

Antiviral assays were performed utilizing TZM-bl cells as previously described [13]. Briefly, plated cells (6×10^3^ cells/well incubated for 48 h, 4×10^3^ cells/well incubated for 24 h, or 8×10^3^ cells/well incubated for 24 h, 100 µl/well, 96 well plates) were treated in triplicate. Original media was removed, and 50 µl treatments were added to cells, which included vehicle only (PBS) or a dilution of SP, semen, SEVI (agitated PAP^286^), agitated PAP^266^, or a combination. Within 5 min of treatment being supplemented, 50 µl of HIV-1 BaL (4 ng p24/ml or 200 pg p24/well) or control media were added to cells. Cells were either treated for 24 h, 3 d, or washed 3 h post-infection as previously described [16].

A repeat of the methodology used to determine the effect of seminal fluid on HIV-1 in infection [16] was carried out as follows. TZM-bl cells were plated (1.4×10^4^ cells/ml incubated 24 h, 280 µl/well, 96 well plate), and treated in triplicate. 40 µl treatments of SP diluted in PBS with or without SEVI or media only controls were pre-incubated with 40 µl of HIV-1 BaL (120 ng/ml or 4.8 ng p24) for 10 m at room temperature. Cells were infected by adding 20 µl of the pre-incubated treatment to the 280 µl of media, thus diluting the treatment and/or virus 15-fold. After 3 h of incubation the treatment media was removed, cells received 200 µl fresh media, and they were incubated for 3 days.

All treated cells were lysed using a Bright Glo luciferase system (Promega, Madison, WI, USA), and the ability to prevent HIV-1 infection was measured as a percent reduction in luciferase (relative light units or RLU) compared to the positive viral control (media and virus only). Metabolic activity of the cells was verified by a tetrazolium-based (MTT) assay according to manufacturer's instructions (R&D Systems, Minneapolis, MN, USA), while cytotoxicity was measured using the standard trypan blue dye exclusion assay.

PM1 cells (1.5×10^5^/0.1 ml) and PBMCs (5×10^5^/0.1 ml) were treated with SEVI with or without SP, and infected with HIV-1 BaL (200 pg p24/0.1 ml) for 2 h. Cells were then washed, and resuspended in fresh media with sample treatments for 5–7 days. Supernatants were collected on alternate days, and cells in culture were resuspended in sample or media alone. To ensure cell viability, standard trypan blue dye exclusion assays were performed. To quantify viral inhibition, the amount of p24gag was measured in cell supernatants with an ELISA (PerkinElmer, Waltham, MA, USA).

### PAP peptide incubations with SP and proteases

SP with or without a broad spectrum Protease Inhibitor (PI) cocktail containing, 4-(2-aminoethyl)benzenesulfonyl fluoride (AEBSF), pepstatin A, E-64, bestatin, leupeptin, and aprotinin (Sigma) was incubated with PAP^286^ or PAP^266^ for 1–24 h at 300 rpm and 37°C. Serial dilutions of whole SP (1%–1∶3200) were incubated with PAP^286^ for 24 h at 1400 rpm and 37°C. Semen derived PSA and PAP were obtained from Sigma, trypsin was obtained from Difco Laboratories (Detroit, MI, USA), and prostasin and matriptase were produced as previously described [26], [27]. The proteases PSA, prostasin, trypsin, and matriptase were diluted with sterile PBS to a final concentration of [1 µM] and incubated with PAP^286^ [54 µM] for 24 h at 300 rpm and 37°C. A series of PSA concentrations (1.5 µM–0.125 µM) were incubated with PAP^286^ (54 µM), and were agitated at 300 rpm for 24 h at 37°C. Whole PAP was resuspended in sterile PBS to [10 µM]. 1% SP was incubated with PAP at a final concentration of [2 µM] at 300 rpm and 37°C for timed intervals of 3, 6, 12 & 24 h. All incubation sample tubes were pulsed briefly in a microcentrifuge, and immediately stored at −20°C. Samples were electrophoresed on mini-16% Tricine-SDS gels, and stained.

### Identification of protease cleavage products

Digested peptide fragments from protease incubations underwent MALDI-TOF/TOF MS/MS analysis using the Model Ultraflex III mass spectrometer (Bruker Daltonics, Billerica, MA, USA) equipped with LIFT capability. Samples were desalted using C18 ZipTips (Millipore, Bedford, MA, USA) and were analyzed in positive reflector mode. External calibration was performed using a mass standards kit for proteomics analyzer (Applied Biosystems). Samples also underwent analysis via nano-LC ESI-TOF MS/MS using the maXis ESI-Q-TOF (Bruker Daltonics) mass spectrometer online with the Dionex model U3000 nanobore HPLC [28]. Data were analyzed with the Sprot database using the Bruker ProteinScape program version 2.1 and Mascot program version 2.2 with enzyme setting to semiTrypsin.

### Statistical analyses

The antiviral, metabolic, and cytotoxicity experiments were performed at least three times, and each of the assays were performed in triplicate or quadruplicate. For the TZM-bl antiviral assays the infected vehicle-only controls were averaged and set as 100% infection. For the PM1 and PBMC antiviral assays, p24 ELISA quantification established the infected vehicle-only control as 100% infection. Metabolic and cytotoxicity assays compared results to the vehicle-only control, calculating variations as a percentage of the baseline. Individual treatments were analyzed by either one-way ANOVA with Tukey's multiple comparisons post-test or two-tailed unpaired t-test.

## Results

### PAP^286^ amyloid fibril formation is inhibited by seminal plasma

**Figure 1:**
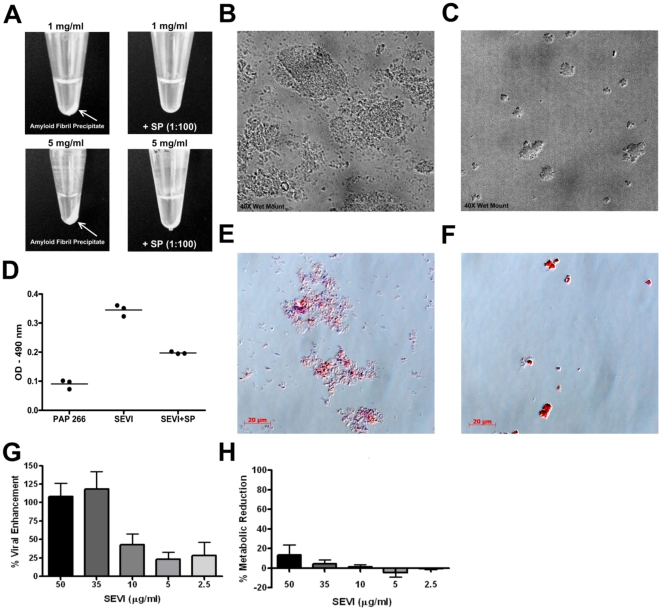
SEVI amyloid fibril formation is inhibited by SP. The synthetic PAP^286^ peptide was agitated at concentrations of 1 mg/ml and 5 mg/ml with or without whole SP (1%) for 36 h at 1400 rpm at 37°C. (A) Sample tubes were centrifuged briefly at 10,000 rpm, and each photographed at the same angle. The 5 mg/ml samples without SP (B) and with SP (C) were vortexed briefly and 20 µl was wet mounted on a slide. (D) Agitated 5 mg/ml PAP^266^, 5 mg/ml SEVI, or SEVI+1% SP were stained with Congo red and measured at 490 nm with a spectrophotometer. Images of stained 5 mg/ml SEVI without SP (E) or with 1% SP (F), were captured at 40x magnification with phase contrast filters under white light using the Axiovert 200 M microscope and Axiovision 4.5 software. (G) TZM-bl cells (6×10^3^ cells/well incubated for 48 h) were treated with serial dilutions of SEVI and infected with HIV-1 BaL (200 pg p24/well). Results are presented as a percent enhancement of viral infection compared to an infected, vehicle-only control. (H) Identically treated cells were also subjected to MTT metabolic assays, presented as percent reduction in cellular metabolism when compared to cells treated with vehicle alone. All presented experiments were performed at least 3 times, and error bars represent SEM.

To confirm our synthetic PAP peptides would form detectable amyloid fibrils *in vitro*, the PAP^286^ and PAP^266^ peptides were agitated separately at either 1 mg/ml or 5 mg/ml according to the established protocol for fibril formation [16]. Only agitated PAP^286^ (“SEVI”) generated turbid solutions and formed an observable precipitate after centrifugation that was concentration-dependent (**Figure 1A**). In the presence of 1% SP (v/v), generation of precipitate was either totally (1 mg/ml SEVI) or partially (5 mg/ml SEVI) inhibited (**Figures 1A**). To visualize the amyloid fibrils, samples were homogenized or stained with Congo red, wet mounted on a slide, and viewed under phase contrast at 40x magnification. Several random field images were taken, and SEVI alone (5 mg/ml) (**Figures 1B & 1E**) revealed numerous fibrillar clusters, while the addition of 1% SP (**Figures 1C & 1F**) substantially reduced fibril formation. The fibril formation comparisons among SEVI, SEVI with 1% SP, and agitated PAP^266^ are demonstrated with the OD values from the Congo red stained samples (**Figure 1D**). To confirm that SEVI fibrils expressed HIV-1 enhancing activity, SEVI was added at various concentrations to TZM-bl cells with R5 strain HIV-1 BaL for 24 h (**Figure 1G**). SEVI demonstrated enhancement of HIV-1 in a dose-dependent manner. To ensure cell viability, MTT assays were run in parallel to the infection assays, and showed no adverse effects to cell metabolism (**Figure 1H**). These studies confirmed that our synthetic PAP peptides form active amyloid fibrils *in vitro*, and that diluted human SP can inhibit amyloid fibril formation.

### The HIV-1 enhancing activity of SEVI is inhibited by seminal plasma

**Figure 2:**
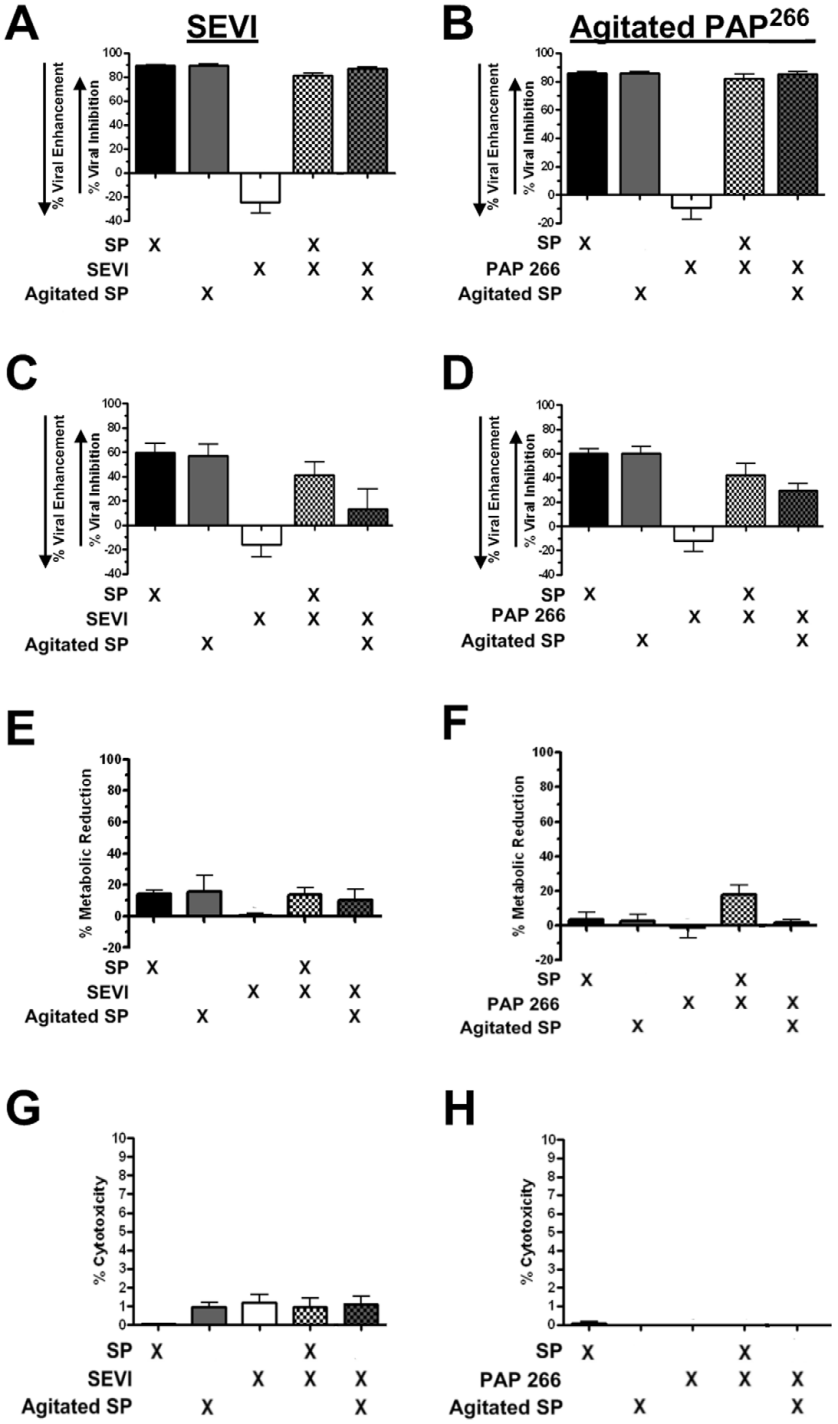
Agitated PAP peptides do not significantly inhibit the anti-HIV-1 activity of SP. Whole SP, synthetic PAP^286^, or synthetic PAP^266^ were agitated at 37°C to promote fibril formation. TZM-bl cells (6×10^3^ cells/well incubated for 48 h) were then treated with either PBS, unagitated SP, agitated SP, agitated PAP^286^ (SEVI, Panel A), agitated PAP^266^ (Panel B), or combinations of agitated peptides and SP, and infected with HIV-1 BaL (200 pg p24/0.1 ml) for 24 h (A & B). SP preparations were administered at a final concentration of 1% (v/v) when diluted with cell media and virus. PAP peptides were administered to cells at a final concentration of 35 µg/ml. (C & D) TZM-bl assays were performed as in A–B except that the cells were washed 3 h post-infection. Results for A–D are presented as % inhibition or enhancement of viral infection compared to the cells infected in the presence of PBS alone. Identically treated TZM-bl cells were also subjected to MTT metabolic assays (E & F) as described in Fig. 1, and trypan blue cytotoxicity assays (G & H) in which % cytotoxicity was calculated from non-viable versus viable cell counts for each treatment condition. All experiments were performed at least 3 times. Error bars represent SEM.

In order to determine if the HIV-1 enhancing activity of SEVI is affected by SP, TZM-bl cells (plated at 6×10^3^ cells/well and incubated for 48 h) were infected with HIV-1 BaL for 24 h in the absence or presence of agitated PAP^286^ (**Figures 2A, C, E & G**) and agitated PAP^266^ (**Figures 2B, D, F & H**). Note that since fibrils formed only with PAP^286^ peptides, but not PAP^266^ peptides, the term “SEVI” applies only to agitated PAP^286^ peptides. In specified conditions, whole SP was also agitated to determine if SEVI fibrils would form and exhibit HIV-1 enhancing activity. SP was utilized at a final concentration of 1% (v/v) to mitigate cytotoxic effects. Treatments with SEVI (**Figure 2A**) and to a lesser extent agitated PAP^266^ (**Figure 2B**) revealed HIV-1 enhancing activity at physiological concentrations (35 µg/ml), but these enhancing effects were negated when combined with agitated or non-manipulated SP. We then repeated these antiviral assays, but instead washed the cells with PBS and replaces with media alone 3 h post-infection as reported in [16]. As shown in **Figures 2C & D**, the trends remained as compared to the 24 h treatment (**Figures 2A & 2B**). Moreover, we performed certain experiments in the absence of FBS as reported in [16], which also revealed that SP could reduce the proviral activity of SEVI and PAP^286^ (data not shown). To verify cell viability, MTT assays were run in parallel to all antiviral assays, and revealed no significant adverse effects to metabolic activity of cells treated for 24 h (**Figures 2E & 2F**) or washed after 3 h (data not shown). For further confirmation, trypan blue assays were also performed in parallel to the infection assays at 24 h of treatment (**Figures 2G & 2H**) and revealed a lack of cytotoxicity.

In the results above, SP exhibited antiviral activity as previously seen in our work [13], but in contrast to the results demonstrated by others using subtle differences in methodology [16]. Therefore, to determine if the difference in SP activity was influenced by methodology of treatment, the methods used to determine the effect of seminal fluid on HIV-1 infection were followed precisely as described previously [16]. Minimally manipulated SP (0.4%, 2%, 10%) was tested in parallel with antibiotic supplemented and filtered SP “SP+(Ab)”, with or without SEVI, such that the final concentrations of SP were (0.026%, 0.113%, 0.66%) (**Figure S1**). While there was no significant reduction in metabolic activity, viral infection was still inhibited by all treatments containing SP at the final concentration of 0.66%, similar to the 1% final concentration of SP used in the current manuscript and in our report [13]. Of note, minimally manipulated SP alone exhibited antiviral activity at every tested concentration, while filtered SP+(Ab) and filtered SP+(Ab) containing SEVI both exhibited HIV-1 enhancing activity. Importantly, these results suggest that viral enhancing and inhibitory activities are concentration-dependent, and that the process of filtration may interfere with certain antiviral components of SP.

We next explored the effect of cell density and SP preparation on antiviral activity. For a 24 h infection period (**Figure S2**), cells seeded at 4×10^3^ cells/well and 8×10^3^ cells/well were treated with either Pre-SP, Post-SP or Semen at 0.4%, 2%, or 10% (final concentrations as in [16]), and infected with a final concentration of 2 ng/ml HIV-1 BaL. The overall antiviral activity of the Pre-SP, Post-SP, and Semen treatments exhibited similar trends between the differing cell densities. However, when the same experiment was extended to a 3 d infection period (**Figure S3**), as performed in reference [29], a significant increase in cytotoxicity was observed.

**Figure 3:**
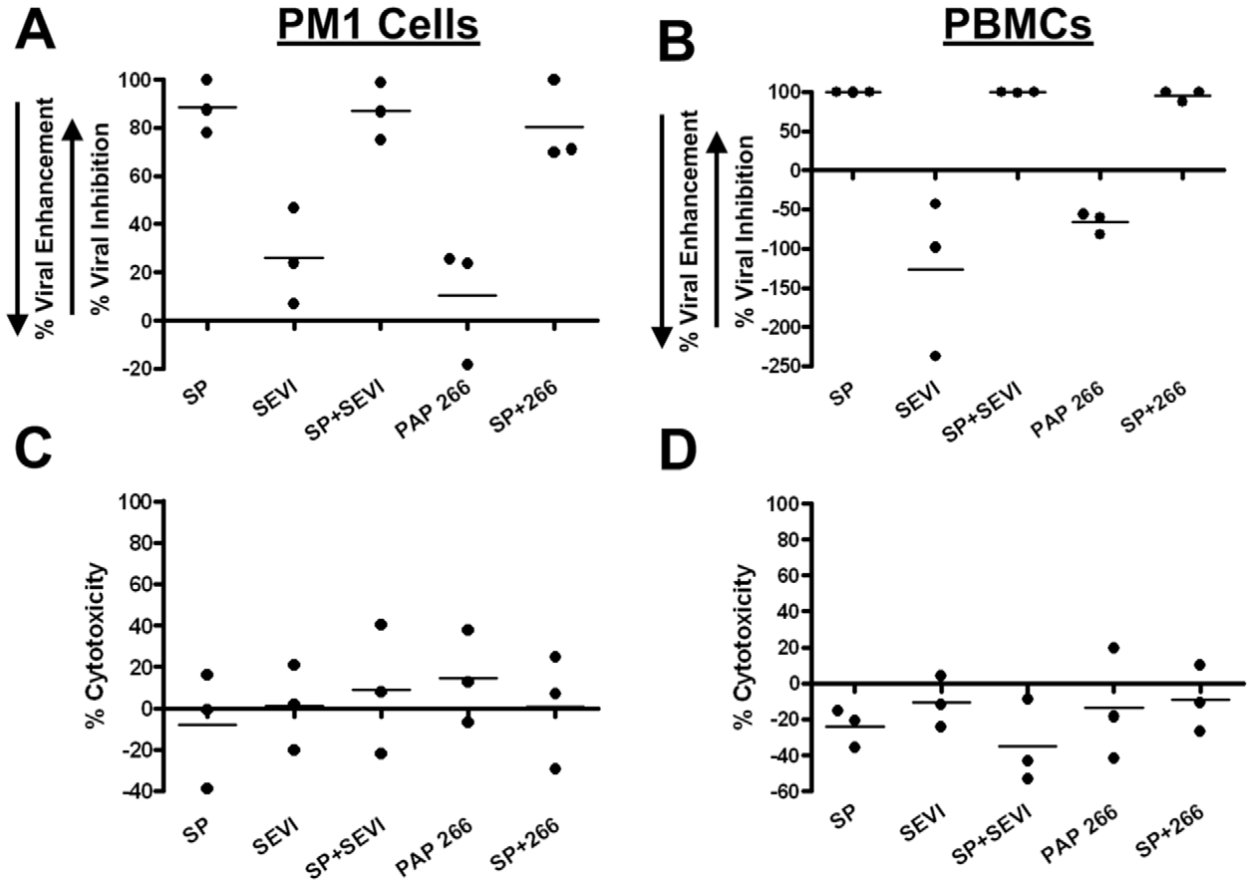
Agitated PAP peptides do not significantly alter SP inhibition of HIV-1 release. (A) PM1 cells and (B) PBMCs were infected with HIV-1 BaL (200 pg p24/0.1 ml) in the presence of whole SP (1%), SEVI, agitated PAP^266^ (35 µg/ml), or SP (1%) and agitated PAP peptides (35 µg/ml) combined, as well as a vehicle-only control. Five days (PM1 cells) and seven days (PBMC's) post-infection, supernatant was collected to quantify p24 release by ELISA. Data are presented as a percent inhibition of infection in relation to the infected vehicle-only control. Treated PM1 cells (C) and PBMCs (D) were also analyzed by trypan blue exclusion for assessment of cytotoxicity. All graphs represent, n = 3.

In order to validate our findings shown in **Figure 2**, infection assays were performed on PM1 cells (**Figures 3A & 3C**) and PBMCs (**Figures 3B & 3D**) by measuring viral propagation over a multi-day time course. Agitated PAP^266^ and SEVI were added to cells at a final concentration of 35 µg/ml with or without 1% SP. PBMC infections confirmed HIV-1 enhancing activity of SEVI alone, while both assays confirmed significant antiviral activity of SEVI spiked with SP. No appreciable cytotoxicity to the cells was detected by trypan blue assays (**Figure 3C & 3D**). Collectively, these results indicate that antiviral activity of SP is retained in the presence of SEVI.

### Seminal plasma naturally degrades PAP peptides over time

**Figure 4:**
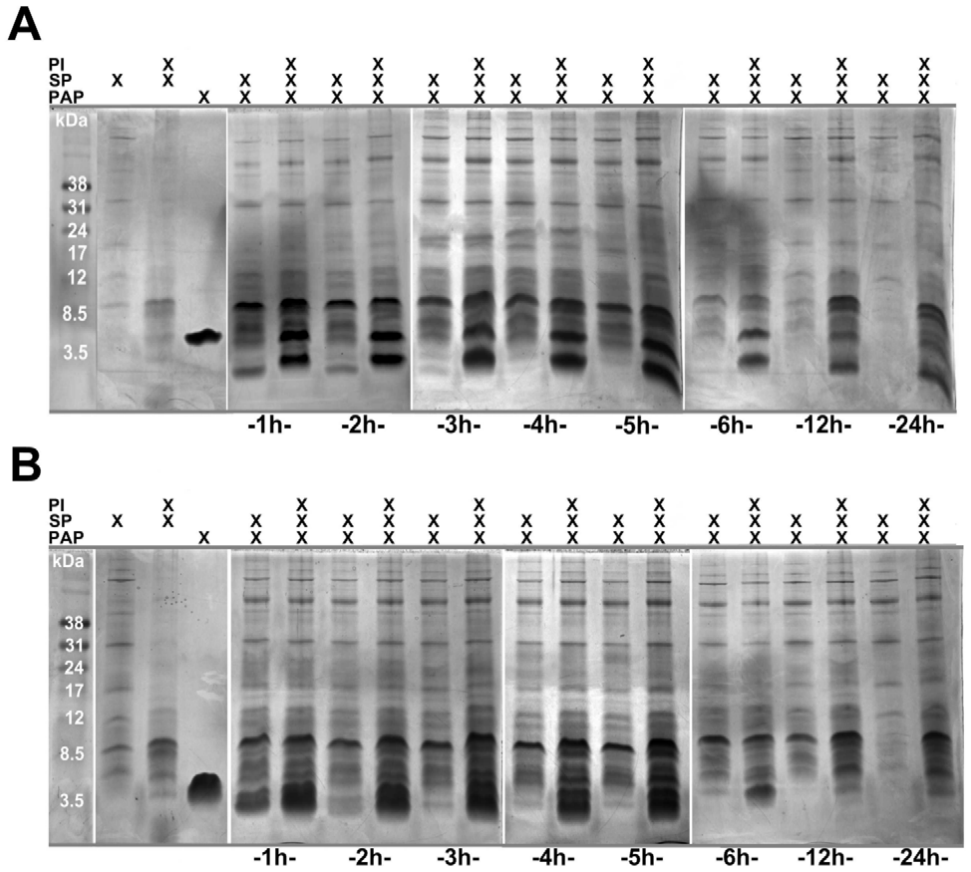
PAP peptides undergo degradation by SP within hours. Purified PAP peptides were incubated with either whole SP, SP spiked with a broad spectrum protease inhibitor (PI), or PBS, and incubated at 300 rpm, at 37°C, for the indicated durations. (A) Three volumes of PAP^286^ (1 mg/ml) were incubated with one volume of whole SP, and 2 µl of samples were added to gels. (B) PAP^266^ (1 mg/ml) was similarly incubated with whole SP, and 4 µl of samples were added to the gels. All samples were electrophoresed on Tricine-SDS-gels, and stained with Coomassie blue.

We previously demonstrated that SP naturally degrades native proteins over extended periods of time [13]. Since the proviral activity of SEVI and agitated PAP^266^ were significantly altered by SP, we aimed to determine if PAP was susceptible to intrinsic degradation. Whole PAP [2 µM] was incubated with 1% SP for varying time periods at 300 rpm and 37°C, electrophoresed on a Tricine-SDS polyacrylamide gel, and silver-stained (**Figure S4**). The majority of the PAP protein was degraded within 12 h of incubation, indicating the susceptibility of whole PAP to SP proteases. Note that the PAP protein alone did not demonstrate any observable self-cleavage (data not shown). Next, SP with or without a protease inhibitor (PI) cocktail was incubated with either PAP^286^ (**Figure 4A**) or PAP^266^ (**Figure 4B**) for set increments of time at 300 rpm and 37°C. Tricine-SDS electrophoresis revealed that PAP^286^ and PAP^266^ underwent partial degradation within 1 h, and complete degradation within 3 h. Samples containing the PI exhibited preservation of the PAP peptides over time, indicating that SP proteases were capable of degrading PAP.

**Figure 5:**
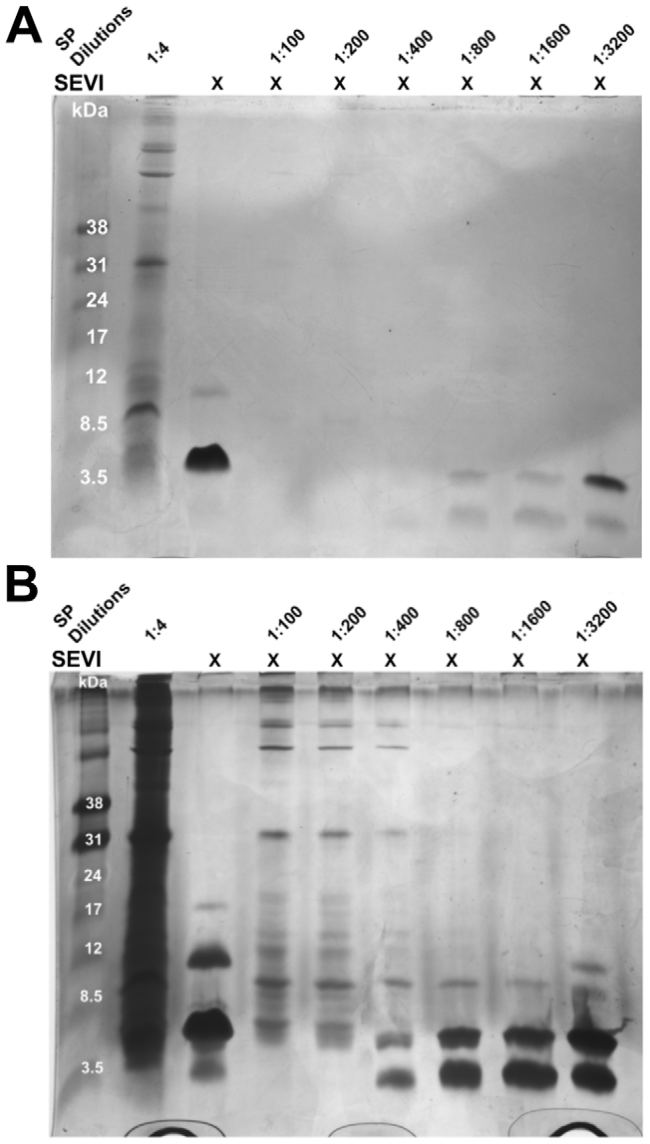
SP can degrade PAP^286^. Freshly resuspended PAP^286^ (1 mg/ml) was incubated with serial dilutions of whole SP at 1400 rpm for 24 h at 37°C. Samples were electrophoresed on mini-Tricine-SDS-gels, and visualized with Coomassie blue (A) and then silver stain (B).

Since 1% SP neutralized the activity of SEVI, the catalytic concentration of SP necessary for PAP peptide degradation was investigated. Serial dilutions of whole SP were incubated with PAP^286^ and samples were electrophoresed on Tricine-SDS polyacrylamide gels, and stained with Coomassie (**Figure 5A**) and then silver stain (**Figure 5B**). Partial degradation of PAP^286^ occurred at dilutions of SP as high as 1∶3200. Complete degradation of PAP^286^ was observed at 1∶200 dilutions of SP, and lower. These results indicate that SP contains fast-acting, PAP-degrading proteases in excess.

### Proteases within SP are capable of degrading PAP^286^

**Figure 6:**
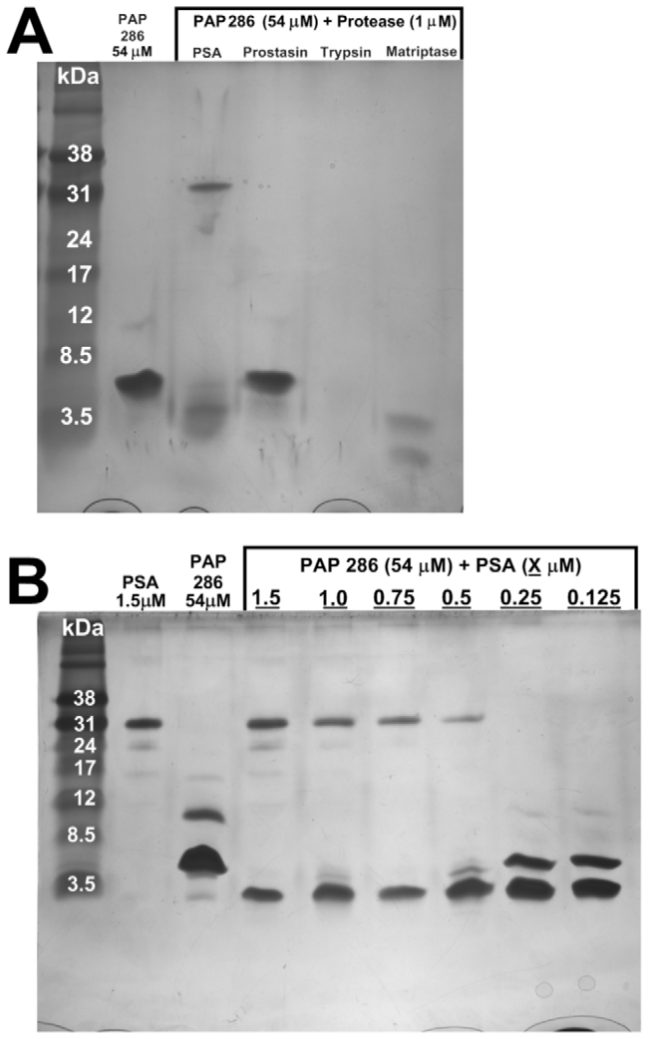
Proteases within SP degrade PAP^286^. (A) PAP^286^ (54 µM; 250 µg/ml) was incubated with various SP proteases (1 µM) at 300 rpm for 24 h at 37°C. 3 µl of each sample were electrophoresed on Tricine-SDS-gels, and silver stained. (B) The predominant SP protease PSA was incubated with PAP^286^ (54 µM) in serial molar dilutions. For each sample, 3 µl were electrophoresed on mini-Tricine-SDS-gels, and silver stained.

Prediction of the protease cleavage sites (Expasy – peptidecutter) revealed trypsin- and chymotrypsin-like cleavage sites within the PAP^286^ peptide, giving rise to the possibility of proteolytic degradation of PAP^286^ by these classes of proteases. In order to determine the specific PAP-degrading proteases in SP, PAP^286^ was subjected to incubation with the SP proteases prostate-specific antigen (PSA), prostasin, and matriptase, and with trypsin as a positive control. PSA exhibits chymotrypsin-like activity, while prostasin and matriptase exhibit trypsin-like activity. The samples were electrophoresed using Tricine-SDS gels to reveal any resulting cleavage products (**Figure 6A**). PSA and matriptase treatments resulted in degradation of the PAP peptide with visible cleavage products, while prostasin did not effectively degrade PAP^286^. Trypsin completely degraded the peptide, with no cleavage products visualized. PAP^286^ was incubated with serial dilutions of PSA (**Figure 6B**), which demonstrated complete PAP^286^ degradation at 0.75 µM. This corresponded to a 1∶72 molar ratio of PSA: PAP^286^ necessary for complete cleavage. Note that even the lowest concentrations of PSA tested (0.125 µM) promoted partial PAP^286^ degradation.

**Figure 7:**
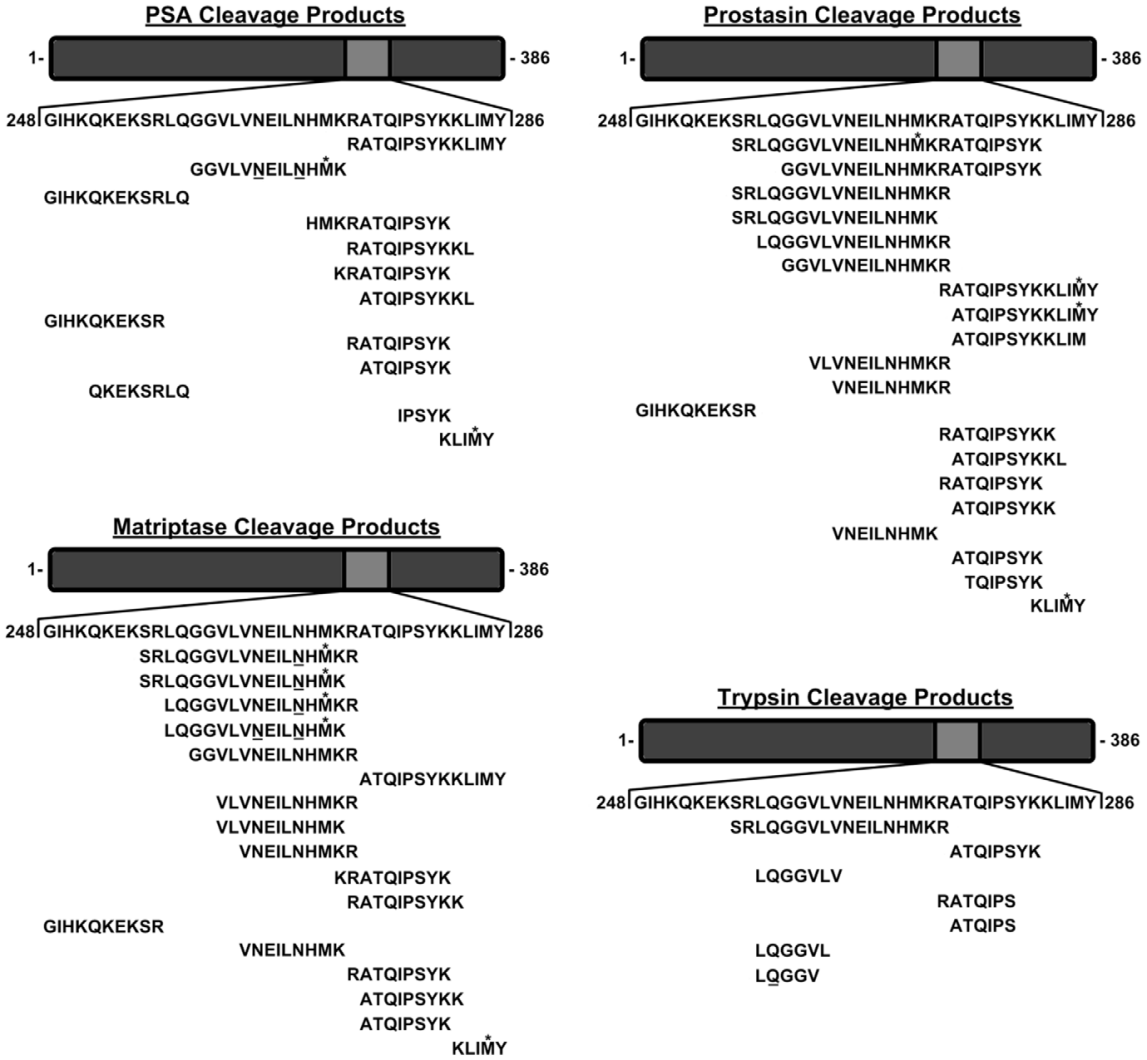
Cleavage products of PAP^286^, following incubation with SP proteases. The incubation products from the SP protease incubations were analyzed via MALDI TOF/TOF MS/MS and nano-LC-MS/MS analysis. The cleavage products for each individual protease were marked under the whole PAP^286^ sequence. Underlined asparagine (N) residues could undergo deamidation, while methionine (M) residues marked with an asterisk (*) could undergo oxidation.

SP was analyzed in triplicate with a PSA-specific ELISA to quantitate the intrinsic amount of this protease, which measured 311 µg/ml (11 µM) in whole SP, consistent with previous reports [7], [30]. Cleavage products from the protease incubations were analyzed via MALDI TOF/TOF MS/MS and nano-LC-MS/MS analysis. All proteases generated detectable cleavage products from incubation with PAP^286^ (**Figure 7**) with some overlap in cleavage sites among the different proteases. Taken together, these results demonstrate the ability of multiple SP proteases to cleave PAP^286^ to various degrees, while the complete degradation of PAP^286^ was demonstrated by the most abundant SP protease, PSA.

## Discussion

Our studies confirmed previous reports that SEVI alone was capable of enhancing HIV-1 infection [16], and additionally revealed that the native PAP^286^ peptides and truncated forms (PAP^266^) were also proviral. This may suggest additional situations *in vivo* in which PAP derived peptides could exert activity in the absence of fully elongated amyloid fibrils. However, we also revealed that proteolytic mechanisms within SP could reduce the proviral effects of SEVI and PAP peptides under certain conditions. Moreover, differences in treatment of SP and semen might also affect concentrations of the antiviral cationic peptide components that we have reported [13]. *In vivo*, both pro- and antiviral situations could easily be explained by heretofore unknown donor-to-donor differences in PAP peptide concentration, protease concentration, and other factors that might affect the pro- and antiviral activity of SP directly or indirectly.

It is interesting to note that the ability of PAP-derived peptides to form amyloid fibrils is a common characteristic for many peptides and proteins given the correct conditions and time [31]. Fibril formation follows a model nucleation-dependent elongation mechanism, initiated by a lag phase for nucleus seeding [32]. When tested at a concentration 57-fold higher than the 35 µg/ml physiological concentration, SEVI exhibited a lag phase of ∼10 h [33]. Since concentration of the purified peptide plays a significant role in fibril formation, the spontaneous formation of SEVI from purified PAP^286^ observed in previous studies may be a prime example of this, due to supraphysiological stock concentrations (i.e. 10 mg/ml) used for fibril formation [16]. Without agitation, it was found that fibril formation at lower concentrations of PAP^286^ may not occur or would require an exponentially longer lag phase time [33]. Considering the lack of intense agitation post-ejaculation *in vivo* and the significantly lower physiological concentration of PAP^286^, the lag phase of SEVI formation might afford ample time for intrinsic inhibitors of SEVI to act.

As we observed, native proteases were responsible for the degradation of whole PAP as well as PAP peptides in the presence of SP. It is important to note that several protease incubation studies we conducted contained a significant excess of PAP or PAP peptides compared to SP or the protease of interest, suggesting that catalytic amounts of proteases in SP are responsible for PAP degradation *in vitro*. In addition, our SP samples contained greater than a 100-fold excess of PSA than would be necessary to degrade PAP^286^. One conclusion might be that the physiological concentration of SP would be sufficient to degrade PAP and PAP peptides *in vivo*. Conversely, it is plausible that an unknown promoter or stabilizer of SEVI formation might exist, which induces the formation of fibrils more rapidly *in vivo*. Notably, mechanisms behind the *in vivo* formation of SEVI warrant additional investigation, given that *in vivo*-formed SEVI fibrils themselves have not yet been reported.

It must be noted that while PSA is the primary candidate for the majority of PAP degradation, we also revealed other SP proteases that could proteolyze PAP^286^. Likewise, proteases in human vaginal fluid, or mucosal proteases activated by the low pH in vaginal fluid might also degrade PAP^286^ or SEVI in the post-coital environment [34]. While it is highly suggestive from our studies that PAP and PAP peptide degradation can occur, the level to which this occurs *in vivo* may vary widely and be one of several reasons why the proviral effect has been reported to vary between individuals [29].

In our study, we have assessed the pro- and antiviral activity of PAP peptides and SEVI, under multiple conditions, many of which reproduced methods and techniques utilized by other groups [16], [25], [29]. In short, the various testing conditions all had minor effects on the pro- and antiviral activities, yet the major finding that SP can abrogate part or all of the *in vitro* proviral activity of SEVI was still substantiated. Syringe-filtered SP could confer HIV-1 enhancing activity under the right conditions, perhaps due to the loss of cationic peptides and proteins as a result of the filtration process. Both the concentration and duration of treatment influenced the overall activity of SP. Variable cell density led to differences in pro- and antiviral activity of SP and semen, as well as differences in cytotoxicity. While methodological nuances may help explain the disparity in data given between research groups, it is unclear which more closely represents the *in vivo* environment of HIV-1 infection.

Previous studies have demonstrated the broad spectrum antimicrobial and antiviral activity of human seminal plasma [12], [13]. The heterosexual transmission of HIV is not efficient, occurring as infrequently as 1 in every 1000 coital acts [35], which might be rationalized in part by the observed antiviral activity of human SP and the cationic antimicrobial and antiviral peptides therein. Still, there are factors in SP that exhibit proviral activity *in vitro*. PAP peptides have been confirmed in semen, and the extent of their HIV-1 enhancing activity varies on an individual donor basis [29]. Our current studies suggest that the formation and activity of SEVI *in vivo* might be challenging in the presence of SP, due to the natural degradation of PAP peptides by several intrinsic proteases within SP. While to date there is a lack of evidence confirming the ability of SEVI amyloid fibrils to form naturally in non-manipulated human SP under physiological conditions *in vivo*, it still remains possible that PAP peptides can generate fibrils *in vivo* and exhibit HIV-1 enhancing activity under the right circumstances. Together, we anticipate that our findings will not only spark intense discussion, but will unlock avenues for continued research on the proviral and antiviral aspects of human SP.

## Supporting Information

Figure S1 **SP manipulation, as well as different infection methods, reveals a contrast in SP antiviral activity.**
TZM-bl cells were plated at 1.4×10^4^ cells/ml with 280 µl/well, and incubated for 24 h. Treatments of 10%, 2% or 0.4% of SP, or SP+(Ab) with or without SEVI (35 µg/ml) or media only controls were pre-incubated with HIV-1 BaL (4.8 ng p24) for 10 min at room temperature. Cells were infected by diluting the treatment and/or virus 15-fold in adding it to the cell media (final 0.66%, 0.113%, and 0.026% SP). After 3 h of incubation the treatment media was removed, cells received fresh media, and they were incubated for 3 d. Inhibition of viral infection is presented as a percent reduction in luciferase activity compared to an infected, vehicle-only control (A). Cells were also subject to MTT metabolic assays (B), presented as the percent metabolic reduction as compared to the negative control. For graphs, n = 3; and error bars represent SEM.
(TIF)

Figure S2 **Infection of two cell densities for 24 h reveals similar activity.**
TZM-bl cells seeded at 4×10^3^ cells/well (A&B) and 8×10^3^ cells/well (C&D) were incubated for 24 h. Cells were treated with a final concentration of 10%, 2% and 0.4% of Pre-SP, Post-SP and semen, and then immediately infected with the BaL laboratory strain of HIV-1 (200 pg p24) for 24 h. Due to limited amount of sample, whole semen was not tested at 10%, and deemed as Not Determined (ND). Inhibition of viral infection was measured as a percent reduction in luciferase activity compared to an infected, vehicle-only control (A&C). Cells were subject to MTT metabolic assays (B&D), given as the percent metabolic reduction as compared to the negative control. For graphs, n = 3; and error bars represent SEM.
(TIF)

Figure S3 **Cell density influences the antiviral and cytotoxicity of a 3 d infection.**
TZM-bl cells seeded at 4×10^3^ cells/well (A&B) and 8×10^3^ cells/well (C&D) were incubated for 24 h. Cells were treated with a final concentration of 10%, 2% and 0.4% of Pre-SP, Post-SP and semen, and then immediately infected with the BaL laboratory strain of HIV-1 (200 pg p24) for 3 d. Due to limited amount of sample, whole semen was not tested at 10%, and deemed as Not Determined (ND). Inhibition of viral infection was measured as a percent reduction in luciferase activity compared to an infected, vehicle-only control (A&C). Cells were subject to MTT metabolic assays (B&D), given as the percent metabolic reduction as compared to the negative control. For graphs, n = 3; and error bars represent SEM.
(TIF)

Figure S4 **Whole PAP is proteolytically degraded by SP over time.**
Whole PAP protein [2 µM] was incubated with whole SP diluted 1∶100 at 300 rpm at 37°C for timed periods. Sample tubes were immediately stored at −20°C when incubations times were ended. 4 µl of each sample were electrophoresed on a mini-Tricine-SDS-gel, and silver stained.
(TIF)
