# Supplementary figures and images for: HIV-1 Enhancing Effect of Prostatic Acid Phosphatase Peptides Is Reduced in Human Seminal Plasma
Source: PLoS One. 2011 Jan 20;6(1):e16285. doi: 10.1371/journal.pone.0016285 (PMC3024420; doi:10.1371/journal.pone.0016285)

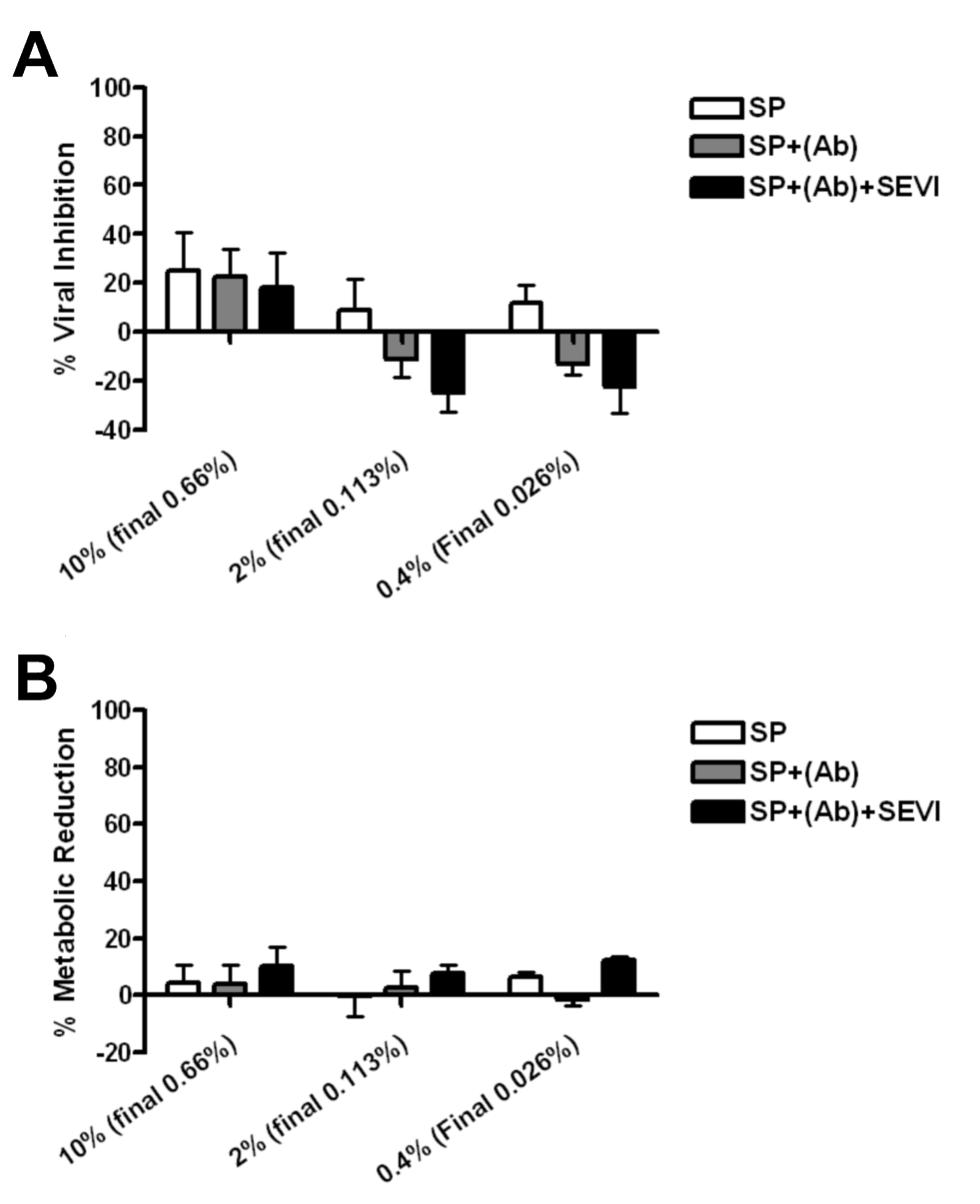

Supplement: Figure S1 — SP manipulation, as well as different infection methods, reveals a contrast in SP antiviral activity. TZM-bl cells were plated at 1.4×10^4^ cells/ml with 280 µl/well, and incubated for 24 h. Treatments of 10%, 2% or 0.4% of SP, or SP+(Ab) with or without SEVI (35 µg/ml) or media only controls were pre-incubated with HIV-1 BaL (4.8 ng p24) for 10 min at room temperature. Cells were infected by diluting the treatment and/or virus 15-fold in adding it to the cell media (final 0.66%, 0.113%, and 0.026% SP). After 3 h of incubation the treatment media was removed, cells received fresh media, and they were incubated for 3 d. Inhibition of viral infection is presented as a percent reduction in luciferase activity compared to an infected, vehicle-only control (A). Cells were also subject to MTT metabolic assays (B), presented as the percent metabolic reduction as compared to the negative control. For graphs, n = 3; and error bars represent SEM. (TIF) [file pone.0016285.s001.tif]

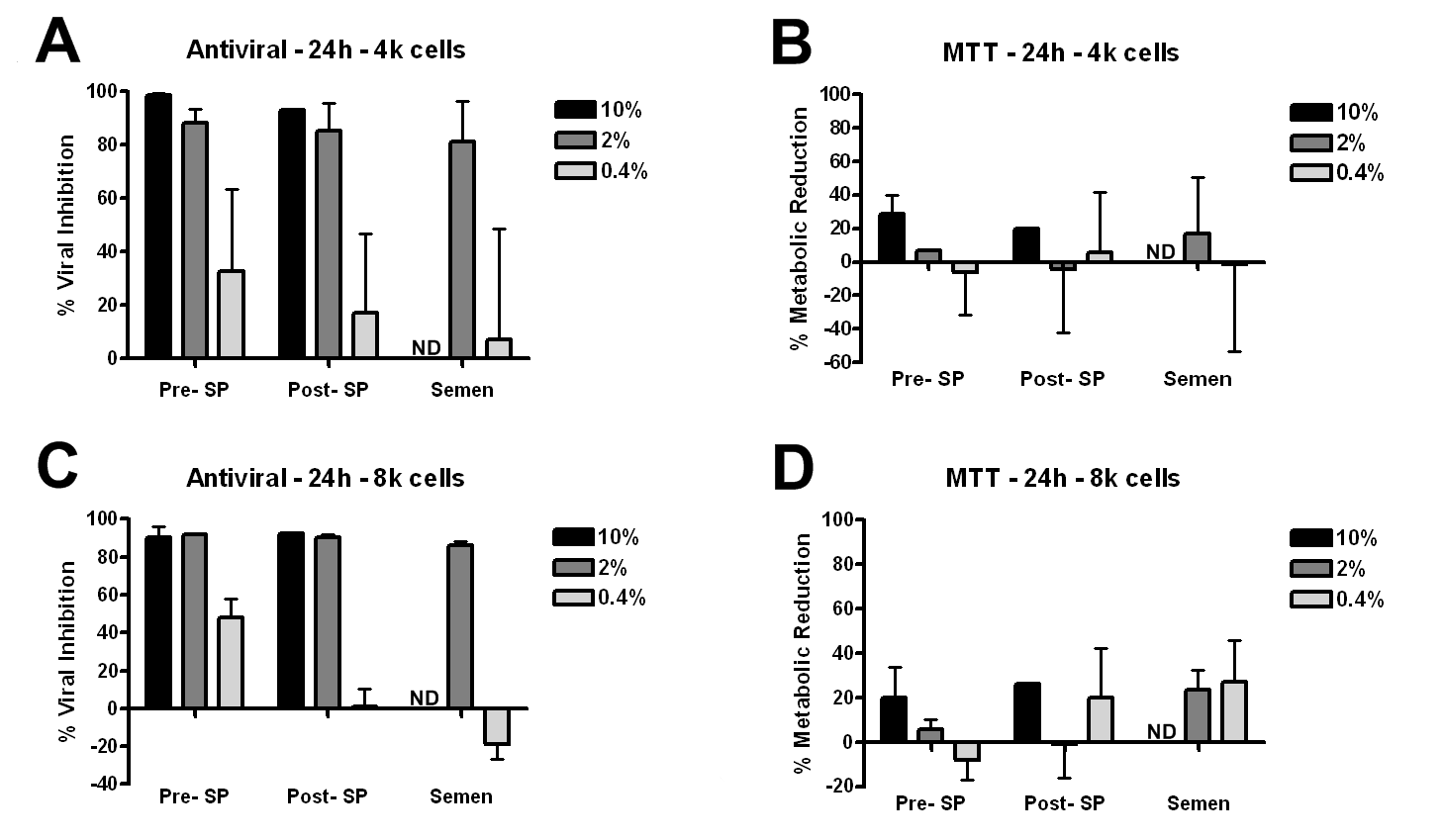

Supplement: Figure S2 — Infection of two cell densities for 24 h reveals similar activity. TZM-bl cells seeded at 4×10^3^ cells/well (A&B) and 8×10^3^ cells/well (C&D) were incubated for 24 h. Cells were treated with a final concentration of 10%, 2% and 0.4% of Pre-SP, Post-SP and semen, and then immediately infected with the BaL laboratory strain of HIV-1 (200 pg p24) for 24 h. Due to limited amount of sample, whole semen was not tested at 10%, and deemed as Not Determined (ND). Inhibition of viral infection was measured as a percent reduction in luciferase activity compared to an infected, vehicle-only control (A&C). Cells were subject to MTT metabolic assays (B&D), given as the percent metabolic reduction as compared to the negative control. For graphs, n = 3; and error bars represent SEM. (TIF) [file pone.0016285.s002.tif]

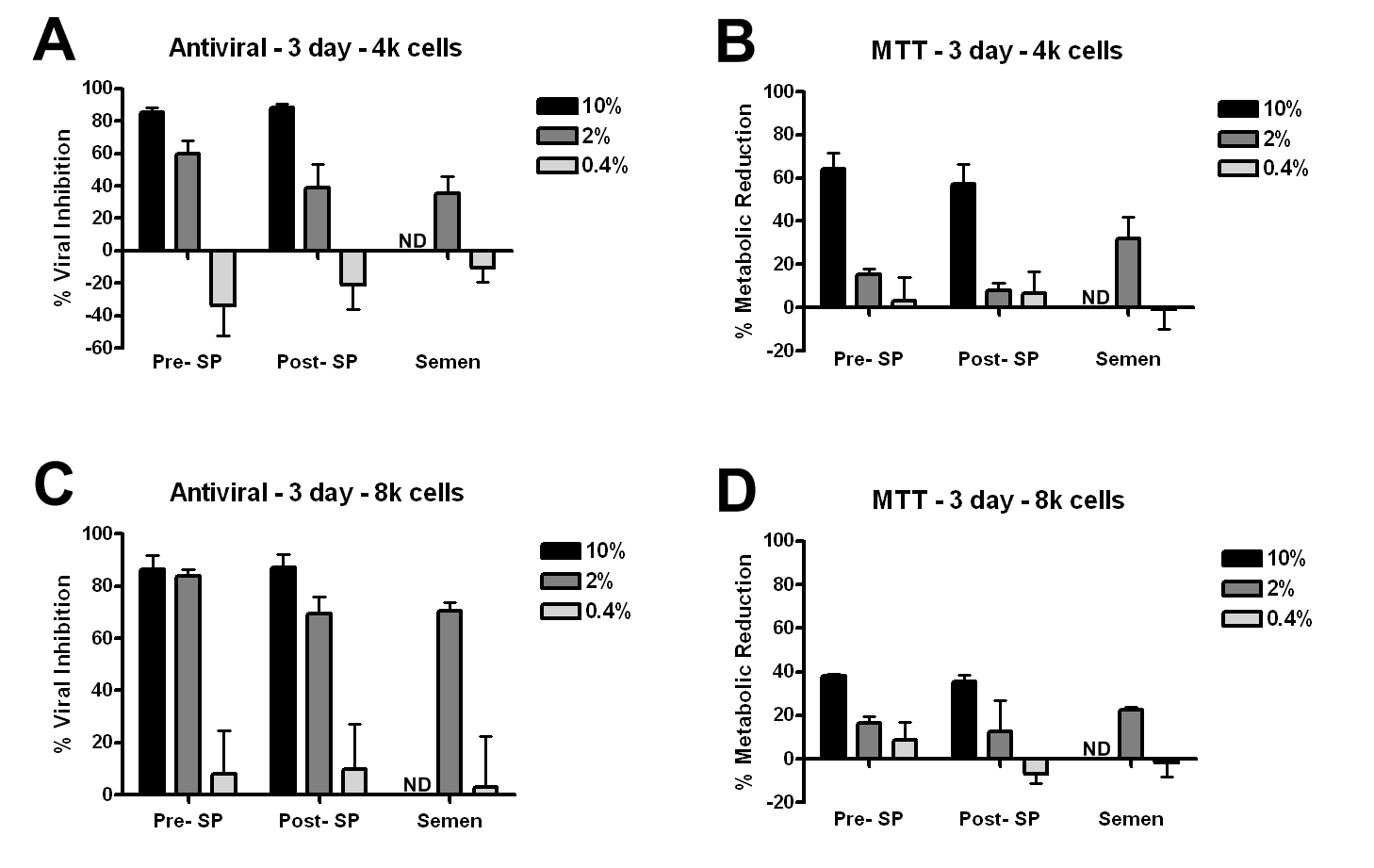

Supplement: Figure S3 — Cell density influences the antiviral and cytotoxicity of a 3 d infection. TZM-bl cells seeded at 4×10^3^ cells/well (A&B) and 8×10^3^ cells/well (C&D) were incubated for 24 h. Cells were treated with a final concentration of 10%, 2% and 0.4% of Pre-SP, Post-SP and semen, and then immediately infected with the BaL laboratory strain of HIV-1 (200 pg p24) for 3 d. Due to limited amount of sample, whole semen was not tested at 10%, and deemed as Not Determined (ND). Inhibition of viral infection was measured as a percent reduction in luciferase activity compared to an infected, vehicle-only control (A&C). Cells were subject to MTT metabolic assays (B&D), given as the percent metabolic reduction as compared to the negative control. For graphs, n = 3; and error bars represent SEM. (TIF) [file pone.0016285.s003.tif]

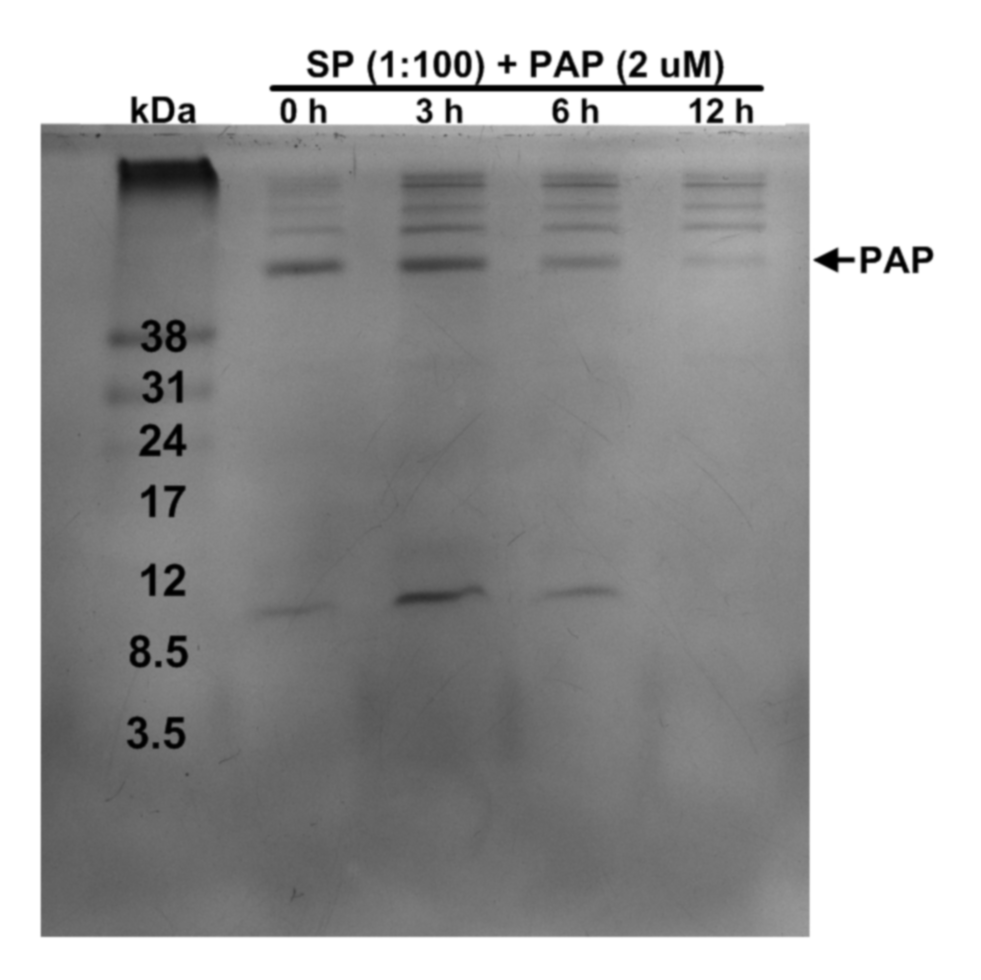

Supplement: Figure S4 — Whole PAP is proteolytically degraded by SP over time. Whole PAP protein [2 µM] was incubated with whole SP diluted 1∶100 at 300 rpm at 37°C for timed periods. Sample tubes were immediately stored at −20°C when incubations times were ended. 4 µl of each sample were electrophoresed on a mini-Tricine-SDS-gel, and silver stained. (TIF) [file pone.0016285.s004.tif]
